# The “beauty in the beast”—the multiple uses of *Priestia megaterium* in biotechnology

**DOI:** 10.1007/s00253-021-11424-6

**Published:** 2021-07-15

**Authors:** Rebekka Biedendieck, Tobias Knuuti, Simon J. Moore, Dieter Jahn

**Affiliations:** 1 Institute of Microbiology and Braunschweig Integrated Centre of Systems Biology (BRICS), Technische Universität Braunschweig, Braunschweig, Germany; 2 School of Biosciences, University of Kent, Canterbury, UK

**Keywords:** *Priestia megaterium*, *Bacillus megaterium*, Recombinant protein production, Vitamin B_12_, Cytochrome P450, Polyhydroxybutyrate (PHB), Plant growth-promoting bacterium, Cell-free transcription-translation

## Abstract

**Abstract:**

Over 30 years, the Gram-positive bacterium *Priestia megaterium* (previously known as *Bacillus megaterium*) was systematically developed for biotechnological applications ranging from the production of small molecules like vitamin B_12_, over polymers like polyhydroxybutyrate (PHB) up to the in vivo and in vitro synthesis of multiple proteins and finally whole-cell applications. Here we describe the use of the natural vitamin B_12_ (cobalamin) producer *P. megaterium* for the elucidation of the biosynthetic pathway and the subsequent systematic knowledge-based development for production purposes. The formation of PHB, a natural product of *P. megaterium* and potential petro-plastic substitute, is covered and discussed. Further important biotechnological characteristics of *P. megaterium* for recombinant protein production including high protein secretion capacity and simple cultivation on value-added carbon sources are outlined. This includes the advanced system with almost 30 commercially available expression vectors for the intracellular and extracellular production of recombinant proteins at the g/L scale. We also revealed a novel *P. megaterium* transcription-translation system as a complementary and versatile biotechnological tool kit. As an impressive biotechnology application, the formation of various cytochrome P450 is also critically highlighted. Finally, whole cellular applications in plant protection are completing the overall picture of *P. megaterium* as a versatile giant cell factory*.*

**Key points:**

• *The use of Priestia megaterium for the biosynthesis of small molecules and recombinant proteins through to whole-cell applications is reviewed.*

• *P. megaterium can act as a promising alternative host in biotechnological production processes.*

## Introduction

**Fig. 1:**
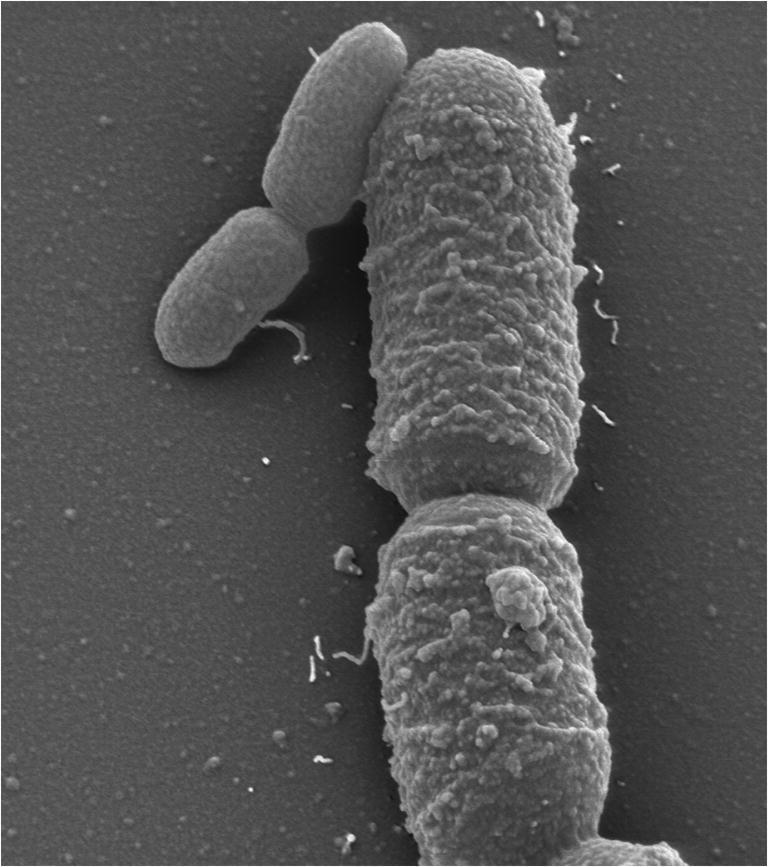
Electron microscope image of *Priestia megaterium* (large cells) and *Escherichia coli* (small cells). *P. megaterium* and *E. coli* were individually grown aerobically in rich medium at 37 °C, mixed in the middle of their exponential growth phases and examined in a field emission scanning electron microscope (FESEM) Zeiss DSM982 Gemini (magnification 6,500-fold). The picture was taken by Manfred Rohde, Helmholtz Centre for Infection Research, Braunschweig, Germany.

Since its discovery in 1884 (de Bary 1884), *Priestia megaterium* (formerly known as *Bacillus megaterium* (Gupta et al. 2020)) provides a powerful cell factory for biotechnology, with numerous patents and applications in industry. The bacterium serves as a model organism for genetic studies and recombinant protein production (Vary 1992; Vary 1994). With its large size of up to 2.5 × 10 μm—“megaterium” literally means “big beast”—it has a significant larger volume compared to that of *Escherichia coli* (Vary et al. 2007) (Fig. 1).

*P. megaterium* is a Gram-positive rod, has a low G+C (~38%) genome, and forms endospores. Its size alone has attracted microbiologists for many years to study its physiology and function including cell division, cell wall biosynthesis, and sporulation (Vary 1994). Now within the last decades, recent advances in modern molecular biology have unlocked its potential for biotechnology. With the expansion of synthetic biology, the many advantages of *P. megaterium* make it an attractive microbial cell factory to rival other model microbes such as *E. coli* and *Bacillus subtilis* (Eisenstein 2016). This review will provide an overview of the diverse potential of *P. megaterium* as a model Gram-positive organism for biotechnological applications including small molecules like cobalamin (vitamin B_12_), the polymer polyhydroxybutyrate (PHB), the production of diverse intra- and extracellular recombinant proteins, whole-cell transformations, and its function as a plant growth-promoting bacterium.

## *Priestia megaterium*—history and genome sequencing

In October 2020, Gupta et al. showed that many *Bacillus* species in addition to the Subtilis and Cereus clade constitute a total of 17 new individual clades based on conserved signature indels (CSIs). They proposed that these clades should be recognized as new genera, with the name *Priestia* gen. nov. for the Megaterium clade containing the former *Bacillus* species *B. megaterium*, *B. abyssalis*, *B. aryabhattai*, *B. endophyticus*, *B. filamentosus*, *B. flexus*, and *B. koreensis* due to two CSIs in the oligoribonuclease NrnB which were uniquely shared by all clade members (Gupta et al. 2020)*.*

*P. megaterium* can be found in diverse habits including honey (López and Alippi 2009), wine (von Cosmos et al. 2017), raw meat (Yucel et al. 2009), fish (Al Bulushi et al. 2010), sea water (Xu et al. 2014), the oral cavity of humans (Al-Thubiani et al. 2018), and most typically plants and soil (Dobrzanski et al. 2018). Consequently, its metabolism is adapted to a variety of different carbon sources including xylose (a byproduct of hemicellulose), glycerol (de Jesus et al. 2016; Korneli et al. 2013; Moreno et al. 2015), disaccharides such as cellobiose, maltose or sucrose (Youngster et al. 2017), and a range of cheap mixed saccharide sources such as sugarcane molasses (Kanjanachumpol et al. 2013).

The first genome sequences of two *P. megaterium* strains (DSM319 and QM B1551) were published a decade ago by Eppinger et al. (2011). Up to now, the full genome sequences including corresponding plasmids of around 20 distinct *P. megaterium* strains are available at the NCBI genome database. Five of these strains lack natural plasmids, while the remaining strains contain up to ten plasmids, consistent with studies already from the early 1980s which found plasmid-less strains to be an exception (Stahl and Esser 1983). The type strain DSM32 (ATCC14581) has been used to conduct basic genetic research. It is also known as the source of the cytochrome P450-BM3 (CYP102A1) (Narhi and Fulco 1986). The plasmid-less DSM319 and its variant MS941, which lacks the gene coding for the major extracellular protease NprM (Wittchen and Meinhardt 1995), are best suited candidates for plasmid-based genetic applications, including the generation of mutants and the recombinant production of proteins (Biedendieck et al. 2011). Strain QM B1551 is used in basic research, especially within the context of sporulation genetics (Manetsberger et al. 2018; Riyami et al. 2019). Strain WSH-002 has been used for co-cultivation with *Ketogulonicigenium vulgare* (Zhang et al. 2010) or *Gluconobacter oxydans* (Lü et al. 2003) to produce vitamin C (Liu et al. 2011). Strain NCT-2, which was isolated from salinization soil from greenhouses, shows high capacity in bioremediation in salinized soil (Wang et al. 2020). Similarly, strain Q3 was described as an endophytic quinclorac-degrading bacterium for bioremediation purposes (Liu et al. 2014), while strains YC4-R4 and TG1-E1 show high salt tolerance as plant growth-promoting rhizobacteria (Vílchez et al. 2018a; Vílchez et al. 2018b). Likewise, strain JX285 acts as plant growth-promoting bacterium, isolated from rhizospheric soil (Huang et al. 2019). Strain SR7 was identified in samples collected from a naturally supercritical carbon dioxide (scCO_2_)-rich environment. SR7 displays resistance to scCO_2_, which is considered to be a promising alternative to classical organic solvents and is already used in in vitro applications (Boock et al. 2019; Freedman et al. 2018).

## *P. megaterium* in biotechnological production processes—from the biosynthesis of small molecules through to whole-cell applications

These different properties clearly highlight the diversity of *P. megaterium* and provide the prerequisite for its diverse applications ranging from the biosynthesis of small molecules, recombinant proteins, and biotransformations to whole-cell bioremediation. Table 1 provides a summary of recombinant proteins and other products produced using *P. megaterium*. Outlined data show that major applications in recombinant protein production with *P. megaterium* rely on a strong protein export system for secretion into the surrounding environment. Overall, proteins with biomedical applications like *Clostridioides difficile* toxins (Yang et al. 2008), protein vaccines (Wang et al. 2018), urokinase-like plasminogen activators (Rygus and Hillen 1991), antibody fragments (Jordan et al. 2007; Lakowitz et al. 2017), and penicillin G acylase (Mayer et al. 2019) constitute major extracellular products. Another important class of proteins is involved in the metabolism of various carbohydrates. It consists of levansucrases (Biedendieck et al. 2007a; Korneli et al. 2013; Malten et al. 2006), α-cyclodextrin glycosyltransferase (Zhou et al. 2012), dextransucrase (Malten et al. 2005b), xylanase (Zheng et al. 2012), glucose dehydrogenase (Rygus and Hillen 1991), β-galactosidase (Rygus and Hillen 1991), and mannitol dehydrogenase (Baumchen et al. 2007), to name a few. Furthermore, enzymes of vitamin B_12_ and heme biosynthesis (Biedendieck et al. 2010; Leech et al. 2003; Mobius et al. 2010; Moore et al. 2013a; Moore et al. 2014), reductive dehalogenases (Payne et al. 2015), and the model green fluorescent protein (GFP) (Biedendieck et al. 2007b; Biedendieck et al. 2007c; Gamer et al. 2009; Stammen et al. 2010a; Stammen et al. 2010b) complete the picture. Finally, the challenging cytochrome P450 enzymes, catalyzing for example stereospecific hydroxylation of steroids or vitamin D_3_, are naturally encoded by different *P. megaterium* genomes and were recombinantly produced using this bacterium (Abdulmughni et al. 2017a; Abdulmughni et al. 2017b; Bleif et al. 2012; Brill et al. 2014; Ehrhardt et al. 2016; Gerber et al. 2015).

**Table 1:** Recombinant proteins and other products produced and secreted with *Priestia megaterium*

Product	Features	Product titer	Reference
Intracellular			
Glucose dehydrogenase (Gdh)	Native promoter of *gdh*	30 U mL^−1^	(Meinhardt et al. 1989)
Glucose dehydrogenase (Gdh)	P_*xylA*_	101.1 U mg_Protein_^−1^	(Rygus and Hillen 1991)
Mutarotase (Mro)		73.7 U mg_Protein_^−1^	
Urokinase-like Plas-minogen activator (Puk)		400 U mL^−1^ per optical density unit	
β-Galactosidase	P_*xylA*_	4,937 mU	(Rygus and Hillen 1991)
	P_*xylA*_^opt.^	5,200 mU	(Hartz et al. 2019)
	Promoter of gene of putative ferrous iron transport protein	6,300 mU	
*Clostridioides difficile* toxin TcdA	P_*xylA*_, size: 308 kDa, his-tag	5-10 mg L^−1^	(Yang et al. 2008)
*Clostridioides difficile* toxin TcdB	P_*xylA*_, size: 270 kDa, his-tag	10 mg L^−1^	
Chimeric protein vaccine Tcd169	P_*xylA*_	n.d.	(Wang et al. 2018)
Chimeric protein vaccine Tcd169Fl	P_*xylA*_	n.d.	
Chloroform reductive dehalogenase	P_*T7*_, his-tag, purification, B_12_-cofactor	180 mg L^−1^ (calculated)	(Jugder et al. 2018)
Reductive dehalogenase RdhA_NP_ plus mutants	P_*T7*_, his-tag, purification, B_12_-cofactor	n.d.	(Payne et al. 2015)
Green fluorescent protein (GFP)	P_*xylA*_, Strep-tag, fed-batch, 52 g_CDW_ L^−1^	274 mg L^−1^	(Biedendieck et al. 2007c)
	P_*sacB*_	7.9 mg g_CDW_^−1^	(Biedendieck et al. 2007b)
	P_*xylA*_^opt.^, fed-batch, 35 g_CDW_ L^−1^, Δ*xylA*-mutant	1.25 g L^−1^	(Stammen et al. 2010a)
	P_*T7*_	50 mg L^−1^	(Gamer et al. 2009)
	P_K1E_	61.4 mg g_CDW_^−1^	(Stammen et al. 2010b)
CbiX	P_*xylA*_, his-tag, purification	n.d.	(Leech et al. 2003)
CbiH_60_	P_*xylA*_, his-tag	n.d.	(Moore et al. 2013a)
HemG	P_*xylA*_, his-tag, purification	n.d.	(Mobius et al. 2010)
Extracellular			
*Clostridioides difficile* toxin TcdB	P_*xylA*_, size: 270 kDa, his-tag, SP_LipA_	n.d.	(Yang et al. 2008)
Keratinase	P_*xylA*_, native signal peptide	186.3 U mL^−1^	(Radha and Gunasekaran 2008)
	P_*amyL*_, native ﻿signal peptide	171.3 U mL^−1^	
Levansucrase SacB	P_*xylA*_^opt.^, glycerol as C-source	520 mg L^−1^	(Korneli et al. 2013)
	P_*sacB*_	4252.4 U L^−1^	(Biedendieck et al. 2007b)
Levansucrase LevΔ773	P_*xylA*_, SP_LipA_	4 mg L^−1^	(Malten et al. 2006)
Levansucrase LevΔ773His	P_*xylA*_, SP_LipA_, his-tag	2.1 mg L^−1^	
Levansucrase StrepLevΔ773	P_*xylA*_, SP_LipA_, strep-tag	2.7 mg L^−1^	
Dextransucrase DsrS	P_*xylA*_, size: 188 kDa, native signal peptide	240 U L^−1^	(Malten et al. 2005b)
α-Cyclodextrin glycosyltransferase	P_*xylA*_, SP_LipA_, codon optimized	8.9 U mL^−1^	(Zhou et al. 2012)
*Thermobifida fusca* hydrolase (TFH)	P_*xylA*_^opt.^, SP_YocH_	7,200 U L^−1^ (7.7 mg L^−1^)	(Stammen et al. 2010a)
Endoglucanase EGI1	P_T7_, 5 different signal peptides, different media	108 mg L^−1^	(Kalbarczyk et al. 2018)
Multimodular cellulose Cel9AT		52 mg L^−1^	
Xylanase	P_*xylA*_, his+strep-tag, purification	304.26 IU mL^−1^	(Zheng et al. 2012)
Thermostable xylanase	P_*xylA*_, his+strep-tag, purification	106 IU mL^−1^	(Sun et al. 2015)
ß-glucosidase (BglZ)	P_*xylA*_	Activity measured in cell extract	(Kurniasih et al. 2014)
Endoglucanase (EglII)			
Fusion protein EglII-BglZ			
*Priestia megaterium* penicillin G acylase (PGA)	P_*xylA*_, SP_LipA_, Δ*xylA*-mutant	41 mg L^−1^	(Yang et al. 2006)
*P. megaterium* PGA	P_*xylA*_^opt.^, native signal peptide, purified from growth medium, crystallization	500 U L^−1^ (20.6 mg_purified enzyme_ L^−1^)	(Mayer et al. 2019)
*Bacillus* species FJAT PGA		550 U L^−1^ (30.2 mg_purified enzyme_ L^−1^)	
*Bacillus thermotolerans* PGA		220 U L^−1^ (15.2 mg_purified enzyme_ L^−1^)	
Hybrid PGAs	P_*xylA*_^opt.^, native signal peptide, purified from growth medium	n.d.	(Mayer et al. 2019)
Single chain PGAs		n.d.	
Chimeric versions of S-layer protein SslA	P_*xylA*_, cell surface display	n.d.	(Knobloch et al. 2012)
Antibody fragment scFV(D1.3) α-lysozyme	P_*xylA*_^opt.^, SP_LipA_, his-tag, micro-bioreactor	14 mg L^−1^	(Lakowitz et al. 2017)
Antibody fragment D1.3 scFab α-lysozyme	P_*xylA*_, SP_LipA_, his-tag	3.5 μg L^−1^	(Jordan et al. 2007)
Whole-cell systems			
Homolog Cbi-enzymes for cobalamin biosynthesis	P_*xylA*_, overexpression of 14 gene *cbi-*operon	Used for cobalamin production (220 μg L^−1^)	(Moore et al. 2014)
HemA	P_*xylA*_	Used for cobalamin production (2.8 μg L^−1^)	(Biedendieck et al. 2010)
HemAXCDBL	P_*xylA*_, integrated upstream of operon	Used for cobalamin production (8.5 μg L^−1^)	(Biedendieck et al. 2010)
Mannitol dehydrogenase (MDH) and formate dehydrogenase (FDH)	P_*xylA*_, 2 gene operon, codon optimized	Whole-cell transformation for D-mannitol production (22 g L^−1^)	(Baumchen et al. 2007)
Cytochrome P450 CYP106A1	P_*xylA*_^opt.^, coproduction with reductase Arh1 and a redox partner	Whole-cell transformation for hydroxylation of 11-keto-β-boswellic acid to 15α-hydroxy-KBA (560.7 mg L^−1^ day^−1^)	(Brill et al. 2014)
Cytochrome P450 CYP106A2	P_*xylA*_^opt.^, coproduction with redox partners AdR and Adx	Whole-cell transformation for hydroxylation of 11-keto-β-boswellic acid to 15α-hydroxy-KBA (560.7 mg L^−1^ day^−1^)	(Bleif et al. 2012)
Cytochrome P450 CYP109A2	P_*xylA*_^opt.^	Whole-cell transformation for the conversion of vitamin D_3_ to 25-hydroxyvitamin D_3_ (54.9 mg L^−1^ day^−1^)	(Abdulmughni et al. 2017a)
Cytochrome P450 CYP109E1	P_*xylA*_^opt.^	Whole-cell transformation for the conversion of vitamin D_3_ to 25-hydroxyvitamin D_3_ (24.5 mg L^−1^ day^−1^)	(Abdulmughni et al. 2017b)
Bovine cytochrome P450 CYP11A1	P_*xylA*_^opt.^, coproduction with redox partners AdR and Adx, codon optimized	Whole-cell transformation for the conversion of cholesterol and analogs (up to 116 mg L^−1^ 48 h^−1^)	(Gerber et al. 2015)
Human cytochrome P450 CYP27A1	P_*xylA*_^opt.^, coproduction with redox partners AdR and Adx, codon optimized	Whole-cell transformation hydroxylation of cholesterol, vitamin D_3_ and 7-dehydrocholesterol (up to 113.14 mg L^−1^ 48 h^−1^)	(Ehrhardt et al. 2016)

## Production of small molecules: cobalamin (vitamin B_12_) in *P. megaterium*

*P. megaterium* is a natural producer of vitamin B_12_ (cobalamin) and has played a prominent role in the study of cobalamin biosynthesis and its industrial production. Cobalamin is a key vitamin for higher eukaryotes, which take it from their diet and require it for B_12_-dependent enzymes (Banerjee and Ragsdale 2003). In nature, cobalamin is only produced by certain species of bacteria and archaea. Derived from the tetrapyrrole family, cobalamin contains a central cobalt ion octahedrally coordinated between four pyrrole nitrogens, a lower ligand (DMB, 5,6-dimethylbenzimidazole) and an interchangeable upper ligand (adenosyl or methyl group). Vitamin B_12_ is officially named cyanocobalamin, where the upper ligand is replaced by cyanide during downstream processing (cyanide extraction), after microbial fermentation. However, the biologically active forms for cobalamin-dependent enzymes are either adenosylcobalamin (coenzyme B_12_) or methylcobalamin (cofactor B_12_). Microbes typically use cobalamin as a prosthetic group for enzymes in primary and secondary metabolism. *P. megaterium* possesses a number of cofactor B_12_ or coenzyme B_12_-dependent enzymes that aid its survival in the environment. This includes ribonucleotide reductase (NrdJ), methionine synthase (MetH), methylmalonyl CoA mutase (MutAB), and ethanolamine lyase (EutBC). In particular, the coenzyme B_12_-dependent EutBC assimilates ethanolamine as a sole carbon and nitrogen source (Roof and Roth 1989; Wolf and Brey 1986).

### *P. megaterium* as a model to study genetics and biosynthesis of cobalamin

**Fig. 2:**
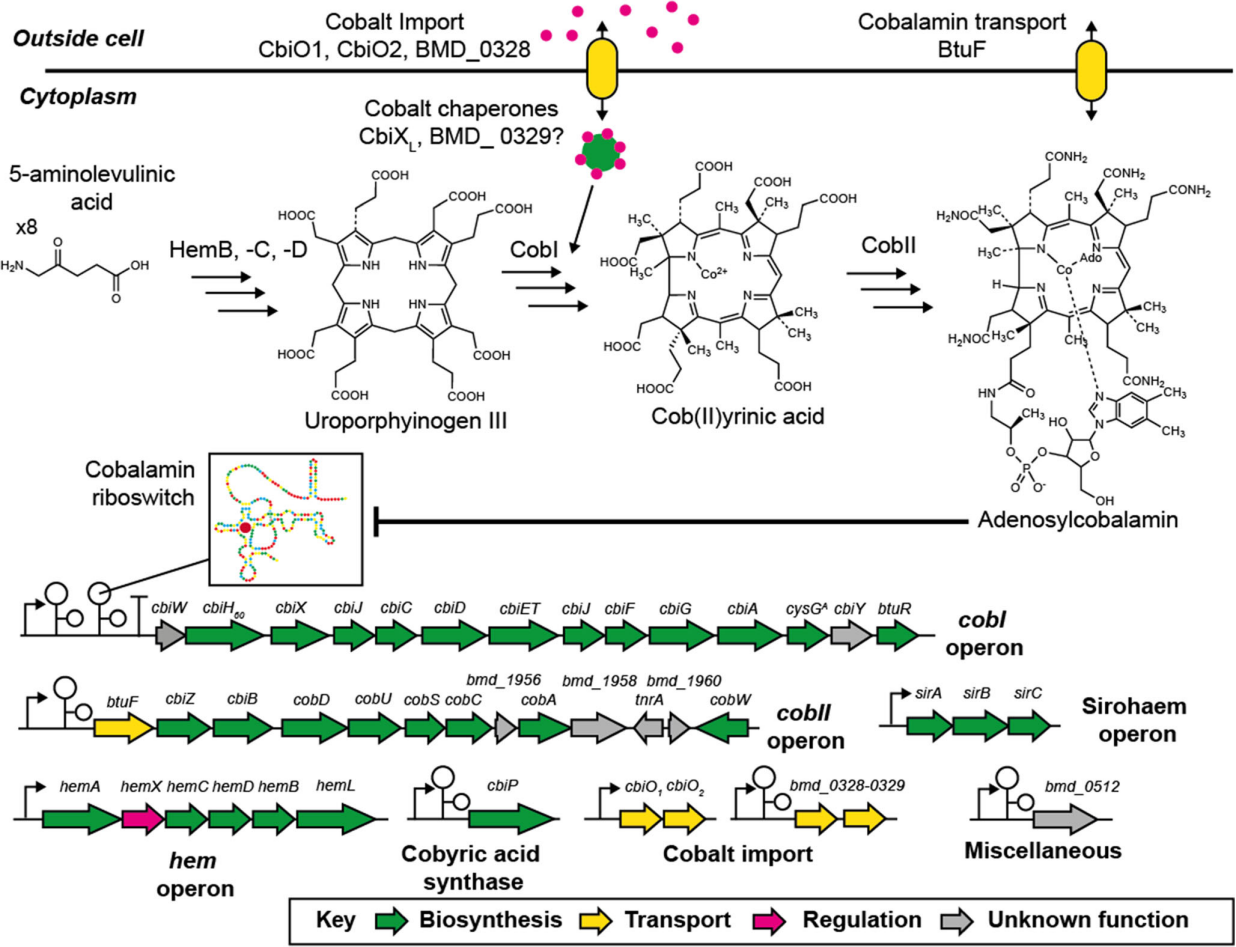
Summary of cobalamin genetics, biosynthesis, and regulation in *Priestia megaterium* DSM319. Upper part: cobalt and cobalamin transporters are indicated in yellow, cobalt in pink, and cobalt chaperon in green. Middle part: summary of cobalamin biosynthesis starting from 8 molecules of 5-aminolevulinic acid. The final product here is shown as adenosylcobalamin which can interact with the cobalamin riboswitches. CobI and CobII indicate all enzymes encoded by the *cobI* and *cobII* operons shown below. Lower part: all genes are represented as colored arrows. Black arrows upstream of the operons/single genes indicate promoters, black “T”s terminators and black stem-loop structures cobalamin riboswitches. All genes clustered in operons or situated on their own are annotated. Hypothetical genes are annotated as open reading frames (*bmd_0000*) as shown in www.megabac.tu-bs.de.

The biosynthesis of cobalamin is complex and requires about 30 enzymes. Therefore, several decades of research has been required to fully decipher the biosynthesis of cobalamin. Originally, *P. megaterium* was identified as a natural producer of cobalamin by studying its growth on ethanolamine, with auxotroph mutants deficient in cobalamin biosynthesis (Wolf and Brey 1986)*.* Since then, it has provided a suitable model to study the biosynthesis of cobalamin. The biosynthesis of cobalamin in *P. megaterium* can be summarized in three stages: stage 1, the synthesis of uroporphyrinogen III; stage 2, assembly of the corrin ring; and stage 3, attachment of the upper and lower axial ligands to the central cobalt ion (Warren et al. 2002). For *P. megaterium* DSM319, the majority of its cobalamin genes are organized into the following biosynthetic operons: *hem* (stage 1), *cobI* (stage 2), and *cobII* (stage 3) (Eppinger et al. 2011) (Fig. 2). An exception to this rule includes the genes encoding for cobalt transport (*bmd_0328*, *cobO*_*1*_, *cobO*_*2*_) and a cobyric acid synthetase (*cbiP*), which are located separately within the genome (Fig. 2). Stage 1 uroporphyrinogen III (uro’gen III) biosynthesis is encoded by the *hem* operon (*hemAXCDBL*) in *P. megaterium*, whose genetic structure is similar to *B. subtilis* (Hansson et al. 1991). Then, cobalamin is built in two stages. For the first stage, *P. megaterium* operates the so-called anaerobic pathway to insert cobalt (Raux et al. 1998; Scott 2001) and build the corrin ring to achieve the first oxygen stable intermediate, cobyrinic acid (Moore et al. 2013b). Then the final steps in cobalamin biosynthesis attach the lower and upper axial cobalt ligands. This final stage is found in many prokaryotic lineages, since it also permits salvaging of vitamin B_12_ precursors (Maggio-Hall and Escalante-Semerena 1999). Distinctly, *P. megaterium* assembles the lower ligand (5,6-dimethylbenzimidazole) through an aerobic pathway for the final stages of its assembly (Collins et al. 2013). Therefore, *P. megaterium* has a customized cobalamin pathway to suit its requirement for molecular oxygen at different stages.

### *P. megaterium* metabolic engineering of cobalamin production

Since cobalamin requires approximately 60 chemical steps for its total synthesis (Battersby 2000), it is essential for biotechnology to make cobalamin through microbial production. *P. megaterium* provides an excellent host for producing cobalamin (Biedendieck et al. 2010; Martens et al. 2002; Moore et al. 2013a; Moore et al. 2013b; Moore et al. 2014). For example, while *P. megaterium* wild-type strains (DSM319, DSM509, and QM B1551) make only low levels (~0.2–1.0 μg L^−1^) of cobalamin in the lab, unpublished industrial strains are believed to reach up to 300 mg L^−1^ (Martens et al. 2002). Since cobalamin biosynthesis is complex (requires 30 enzymes), there are several bottlenecks that limit its production. This includes biosynthesis of precursors, import of cobalt (Fig. 2), feedback regulation, and rate-limiting enzymes. We will discuss how these individual steps can be optimized in *P. megaterium*.

The supply of precursors such as uroporphyrinogen III (uro’gen III) for the main tetrapyrrole scaffold and *S*-adenosyl-L-methionine (SAM) for methylation represents a major bottleneck for cobalamin biosynthesis in *P. megaterium*. For example, glutamyl-tRNA reductase (HemA) is regulated at both the transcriptional and post-translation level (Schobert and Jahn 2002), through a negative feedback mechanism in heme biosynthesis. Overexpression of a proteolysis-resistant *hemA* mutant in *P. megaterium* increases cobalamin levels 11-fold to 2.8 μg L^−1^ (Biedendieck et al. 2010). Furthermore, to increase uro’gen III supply directly, chromosomal overexpression of the uro’gen biosynthesis operon (*hemAXCDBL* operon) increases cobalamin levels up to 8.5 μg L^−1^. Therefore, the supply of 5-aminolevulinic acid (5-ALA) and uroporphyrinogen III is a major limiting factor in cobalamin biosynthesis.

Cobalt is essential for cobalamin biosynthesis (Martens et al. 2002). Crucially, like any transition metal, regulation is required to avoid toxicity. *P. megaterium* has a range of unique regulatory features to control cobalt levels. In *P. megaterium* DSM319, the addition of cobalt (1–10 μM) alone increases cobalamin levels up to 13 μg L^−1^ (Moore et al. 2014). However, cobalt homeostasis and its incorporation into cobalamin biosynthesis is poorly understood. At the enzyme level, the cobaltochelatase CbiX^L^ inserts cobalt into the tetrapyrrole macrocycle and may play a role in cobalt homeostasis (Fig. 2). Overproducing CbiX^L^ in *P. megaterium* DSM509 in the presence of cobalt increases cobalamin levels by 6-fold (Biedendieck et al. 2010). Interestingly, CbiX^L^ has an extended C-terminal domain that harbors a 4Fe-4S cluster and polyhistidine-rich motif (Leech et al. 2003). While the C-terminal extension is not essential for its chelatase activity (Leech et al. 2003), it may regulate or sense cobalt levels. For example, cobalt can substitute iron in Fe-S clusters (Ranquet et al. 2007) and polyhistidine motifs coordinate transition metals. For cobalt transport, *P. megaterium* has two potential cobalt transporters from the *cbiO* ATPase family or a single-component dual cobalt and nickel transporter (*bmd_0328*) (Komeda et al. 1997). Interestingly, *bmd_0328* is part of two gene operons, containing an uncharacterized gene with another shorter polyhistidine motif (HXXXHH) (*bmd_0329*). Both genes are co-localized with an upstream cobalamin riboswitch (Fig. 2), suggesting their role in cobalamin biosynthesis. B_12_ riboswitches are *cis*-regulatory RNA elements that provide tight negative feedback control when cobalamin levels are high by sequestering either the Shine-Dalgarno site or by forming a transcription attenuator. While the role of *bmd_0329* is unknown, overexpression of *bmd_0328* in *P. megaterium* DSM319 leads to growth sensitivity in the presence of cobalt, suggesting increased cobalt import and toxicity (Moore 2011).

Unlike most prokaryotic metabolic pathways, there are no known specific transcription factors to regulate gene expression of cobalamin biosynthesis. Instead, global signals such as molecular oxygen repress cobalamin biosynthetic genes in *Salmonella typhimurium* (Escalante-Semerena and Roth 1987). Intriguingly, overproduction of the global anaerobic respiratory regulator FNR (fumarate and nitrate reductase regulator) in *P. megaterium* DSM509 increased cobalamin synthesis by 4-fold (Biedendieck et al. 2010), suggesting that cobalamin biosynthesis is globally regulated by oxygen. Instead of transcription factors, cobalamin biosynthesis is regulated by cobalamin riboswitches. The *P. megaterium* DSM319 genome contains eight cobalamin riboswitches. This includes genes encoding B_12_-independent enzymes (*metE*, *nrdEF*), complete pathways (*cobI* and *cobII* operons), cobyric acid synthetase (*cbiP*), and cobalt homeostasis genes (*bmd_0328-bmd_0329, bmd_0512* (see above)) (Fig. 2). The *cobI* operon is regulated by a cobalamin riboswitch and transcription terminator and is highly sensitive (nM levels) to cobalamin (Moore et al. 2014). This is not surprising since prokaryotic cells only require trace levels of cobalamin for unrestricted growth. Instead scavenging cobalamin from the environment (Nahvi et al. 2004) is also supported by an ABC transporter *btuF* (located within the *cobII* operon) and an uncharacterized transporter (*bmd_0512*), both co-localized with a cobalamin riboswitch (Fig. 2).

The cobalamin riboswitches represent the major bottleneck in engineering cobalamin biosynthesis in *P. megaterium* (Fig. 2). To bypass this metabolic feedback, the entire *cobI* operon was placed under the control of a constitutive promoter on a multi-copy plasmid. Remarkably, expression in *P. megaterium* DSM319 in the presence of 10 μM cobalt led to major increases in cobalamin levels to 220 μg L^−1^, a 27.5-fold increase over the control strain (Moore et al. 2014).

## Production of biopolymers using *P. megaterium*: polyhydroxybutyrate (PHB)

Polyhydroxyalkanoates (PHAs) are naturally occurring biopolymers synthesized by many microorganisms in response to environmental stress. They are considered to have promising potential to substitute traditional petrol-based plastics, as these so-called bioplastics show similar chemical and physical properties as conventional plastic (Chen 2009; Lu et al. 2009). PHAs were described in 1926 by the French scientist Lemoigne, who observed that *P. megaterium* accumulated polyhydroxybutyrate (PHB), a specific form of PHA, in the cells as distinct granules (Lemoigne 1926). Inside the cells, PHB acts as a storage device for carbon and energy and can be used again when conditions change. The hydrophobic granules are surrounded by a phospholipid monolayer in which a number of specific proteins are embedded, thereby associating with the granules (Jendrossek 2009). For the biological synthesis of PHAs, a variety of different C-sources, even crude waste material like glycerol derived from biofuel production, can be used (de Jesus et al. 2016; Naranjo et al. 2013; Solaiman et al. 2006). The key step in this process, the enzymatic polymerization of hydroxyacyl-coenzyme A (CoA) to PHA and CoA, is catalyzed by a PHA synthase. In 2001, McCool and Cannon identified the genetic organization of the five involved *P. megaterium* genes in two divergent orientated operons consisting of *phaRBC* and *phaQP* (McCool 2001). The *phaC* and *phaR* genes encode the two subunits of the PHA synthase (McCool 2001; Tsuge et al. 2015). Within the heterodimer, PhaC is the catalytic subunit localized with the granules, while PhaR is needed for polymerization (McCool 2001). PhaR from *P. megaterium* should not be confused with PhaR from other organisms like *Ralstonia eutropha*, where the name designates a transcriptional regulator of PHB synthesis (Lee et al. 2004). For the protein PhaB, a NADPH-dependent acetoacetyl coenzyme A reductase function was proposed. PhaB is involved in the supply of (R)-3HB-CoA monomer for the polymerization of PHB (Tsuge et al. 2015). PhaP as a phasin is localized with the granules (McCool and Cannon 1999). These non-enzymatic proteins are commonly found in PHA producers and have been shown to influence the PHA granule morphology and size (Jendrossek 2009). In *P. megaterium* the *phaQ* gene codes for a transcriptional regulator that negatively regulates the expression of *phaP* and *phaQ*. It interacts directly with PHB like PhaR in *R. eutropha*, although it has evolved independently (Lee et al. 2004). To ensure the abundant occurrence of phasin PhaP, but secure the required low level of the regulator PhaQ, Lee et al. speculate that the *phaQ* mRNA, as part of the *phaQP* transcript, is systematically degraded (Lee et al. 2004).

### Optimizing PHB production in *P. megaterium*

To date, there are hardly any attempts described to develop *P. megaterium* toward an increased production of PHB through genetic engineering. In contrast much effort has been placed on optimizing cultivation conditions of environmental isolates of *P. megaterium* to increase PHB production with mainly molasses as carbon source, resulting in almost 70% PHB of cell dry weight (Gouda et al. 2001; Rodríguez-Contreras et al. 2013). For the well-known and genome sequenced strain DSM319, Godard et al. (2020) observed that the PHB content increased 5-fold to almost 30 % of cell dry weight under high salt conditions (Godard et al. 2020). Further, the production of functionalized PHB granules provides an exciting new application for *P. megaterium*. One recent study showed that the mammalian cytochrome P450 CYP11A1 could be immobilized and purified with PHB granules produced in *P. megaterium*, thereby circumventing the problem of low stability of recombinantly produced cytochromes. Here, CYP11A1 was readily localized in the phospholipid monolayer of the PHB granule in its native form verified by denaturing PAGE (Stenger et al. 2018). Another study showed that the IgG binding domain of Protein A from *Staphylococcus aureus* (ZZ domain) could be produced, purified, and presented on PHB granules when fused to PhaC in *P. megaterium*. The isolated functionalized PHB beads were capable of purifying IgG from human serum, thereby proving their functionality (Grage et al. 2017).

## Production of recombinant proteins using *P. megaterium*: intra- und extracellular formation at g/L scale

The Gram-negative *E. coli* represents a well-established and heavily used host for the production and purification of recombinant proteins. However, *E. coli* has some major drawbacks including the presence of endotoxins (LPS) or limitations in the secretion of proteins into the growth medium, which permits easier downstream processing (Lakowitz et al. 2018; Terpe 2006). In contrast, Gram-positive bacteria lack an outer membrane, thereby omitting endotoxins and making protein secretion much more efficient. Many Gram-positives have shown promising potential for recombinant protein production, for example, multiple members of the genus *Bacillus* (Terpe 2006).

**Fig. 3:**
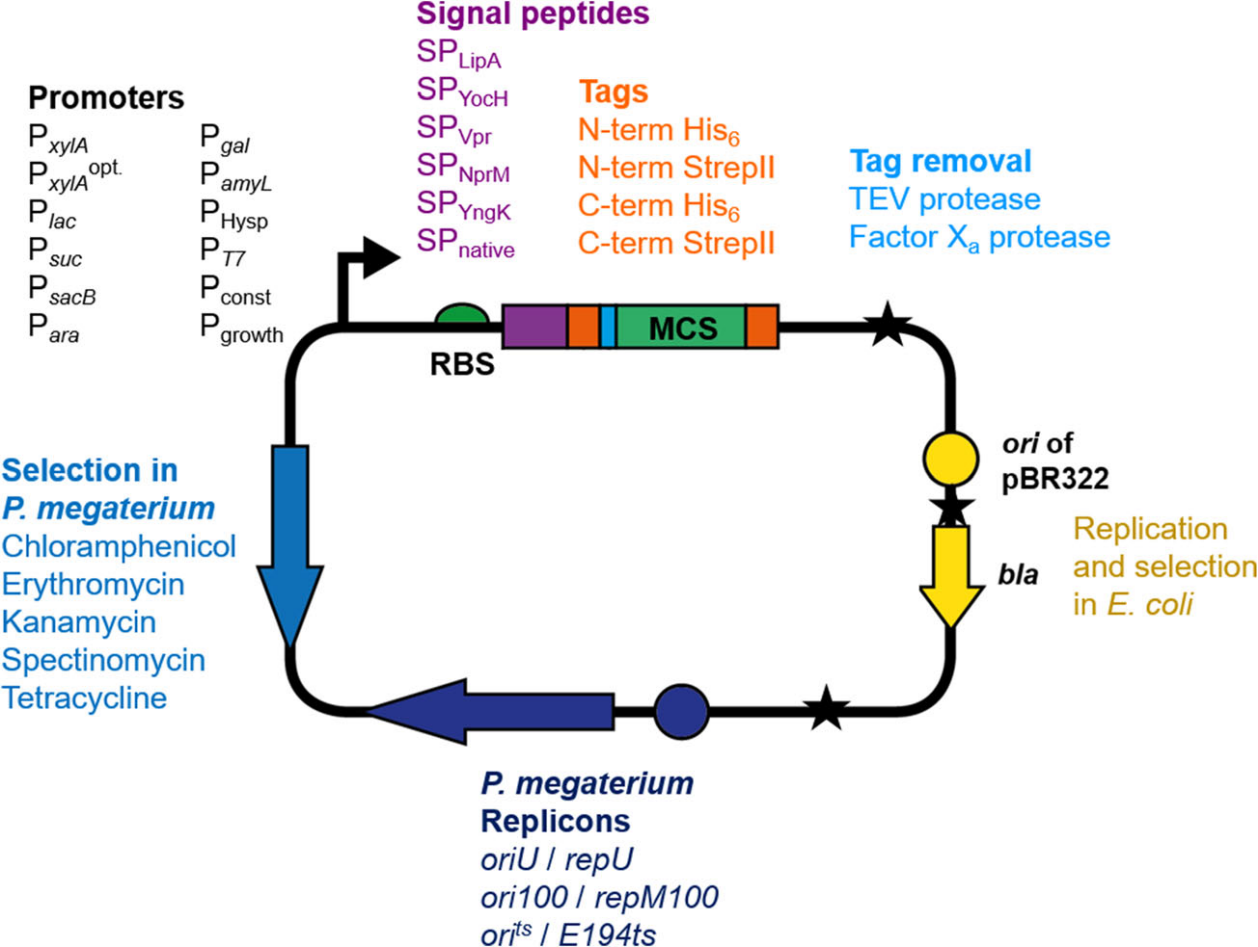
Schematic summary of *Priestia megaterium* plasmids used for the production, secretion, and purification of recombinant proteins. All plasmids are constructed as shuttle plasmids for cloning in *E. coli* (yellow elements) and replication (dark blue, different compatibility classes), selection (blue), and production of recombinant proteins in *P. megaterium*. Suitable promoters (black arrow) are the native (P_*xylA*_) and the optimized (P_*xylA*_^opt.^) xylose-inducible promoter, the lactose inducible (P_*lac*_), sucrose (P_*suc*_, P_*sacB*_), arabinose (P_*ara*_), galactosidase (P_*gal*_), IPTG (P_Hysp_), and starch (P_*amyL*_) promoter, the T7-RNA-polymerase-dependent promoter which is based on a two-plasmid system, and several constitutive (P_const_) and growth phase-dependent (P_growth_) promoters. Genes encoding recombinant proteins can be fused to coding sequences of different signal peptides (purple) of the lipase A (SP_LipA_), the unknown secreted proteins YocH (SP_YocH_) and YngK (SP_YngK_), the natural protease NprM (SP_NprM_), and the serine protease VPR (SP_Vpr_). In addition original signal peptides of the foreign recombinant protein can be used (SP_native_). For purification of intra- or extracellular recombinant proteins, a fusion to N- or C-terminal His_6_ or StrepII tag is possible (orange). N-terminal tags can be removed of using tobacco each virus (TEV) or factor X_a_ protease cleavage (light blue). Black stars indicate stable places for integration of additional genetic elements as tRNAs or genes for co-expression.

In general, *P. megaterium* is renowned for its high stability of recombinant plasmids, even in the absence of selective antibiotics (Radha and Gunasekaran 2008). The production of recombinant proteins using *P. megaterium* is typically performed by plasmid-based gene expression. The majority of these plasmids are based on the *oriU*/*repU* system derived from the pBC16 plasmid, originally found in *Bacillus cereus* (Bernhard et al. 1978; Rygus and Hillen 1991; Rygus et al. 1991), or on the compatible *ori100*/*repM100* system from a plasmid found in *P. megaterium* QM B1551 (Eppinger et al. 2011; Gamer et al. 2009; Stevenson et al. 1998). In addition, the temperature-sensitive *ori*^*ts*^/*E194ts* system from plasmid pE194 is suited for genomic integration experiments (Biedendieck et al. 2010). All plasmids are designed as shuttle vectors enabling all cloning in *E. coli* and subsequent transfer to *P. megaterium* via protoplast transformation (Biedendieck et al. 2011), a technique even suited for new environmental *P. megaterium* isolates (Boock et al. 2019). Standard antibiotics such as tetracycline, kanamycin, chloramphenicol, erythromycin, or spectinomycin can be used as selection markers for *P. megaterium* (Fig. 3). Importantly, all plasmids allow stable replication and production of recombinant proteins in *B. subtilis* or *Bacillus licheniformis* (Lakowitz et al. 2017; Larsen and Bjerga 2018).

### Promoter systems for recombinant protein production in *P. megaterium*

A controlled high-level production of recombinant proteins in *P. megaterium* is based on the native xylose-inducible promoter/repressor system. The promoter P_*xylA*_ is induced in the presence of xylose through a de-repression mechanism based on the inactivation of the repressor XylR via xylose binding. The corresponding gene *xylR* is encoded on the same expression plasmid (Rygus and Hillen 1991; Rygus et al. 1991). The P_*xylA*_*-*based expression system has undergone several systematic optimization steps resulting in intracellular recombinant protein production rates of up to 1 g L^−1^ (Stammen et al. 2010a) and more than 500 mg L^−1^ extracellularly (Korneli et al. 2013). This has resulted in the widely used *P. megaterium* recombinant protein production system, which comprised almost 30 plasmids and four strains and is available for commercial use from MoBiTec (Göttingen, Germany).

Moving on from the xylose-inducible system, a number of alternative promoter systems have recently been studied for protein production, expanding the *P. megaterium* plasmid toolbox (Fig. 3). Among these promoters are homologous sugar-inducible promoters (sucrose, arabinose, galactose, lactose) which were identified from transcriptome analyses. The employment of these systems for recombinant protein production resulted in up to 80 % yield compared to the optimized P_*xylA*_-based system (Biedendieck et al. 2007b; Hartz et al. 2019). Also a heterologous starch-inducible promoter (P_*amyL*_) from *Bacillus amyloliquefaciens* was found less effective compared to P_*xylA*_-based system (Radha and Gunasekaran 2008). Furthermore, the isopropyl-β-D-thiogalactopyranosid (IPTG)-inducible hyper-spank promoter (P_Hysp_) yielded approximately 60 % of recombinant proteins compared to the P_*xylA*_-based system (Boock et al. 2019). As an alternative to these bacterial systems, phage-derived RNA polymerase (RNAP) systems have been successfully applied for protein production in multiple bacteria. For *P. megaterium* the genes coding for the RNAPs from the bacteriophage T7 (Gamer et al. 2009) and the *E. coli* phage K1E (Stammen et al. 2010b) residing on a separate plasmid were expressed under the control of P_*xylA*_. Upon xylose-based gene induction, the phage RNAPs are produced and specifically recognize the corresponding phage promoters driving the target gene expression localized on the second plasmid. The phage promoter-dependent gene expression resulted in up to 10 times more recombinant protein compared to the P_*xylA*_-based system (Gamer et al. 2009; Stammen et al. 2010b).

Beside inducible promoters, a number of constitutive and growth phase-dependent homologous promoters were tested for recombinant protein production. The promoters of the pyruvate dehydrogenase operon (*pdhABCD*) and of genes involved in glycolysis and gluconeogenesis (*fba*, *fbp*, *gap*, *pgc*, *pgi*, and *pgk*) from strain DSM319 yielded up to 75% of recombinant proteins compared to optimized P_*xylA*_-based system (Moore et al. 2018). In addition, some growth phase-dependent promoters were identified with slightly increased protein yields as the P_*xylA*_-based optimized system (Hartz et al. 2021).

### Secretion of recombinant proteins with *P. megaterium*

As mentioned above, *Bacillus* excels as a good secretion host for proteins. Ninety percent of all extracellular *Bacillus* proteins are secreted by the secretion(SEC)-dependent pathway (Tjalsma et al. 2004; Tjalsma et al. 2000) guided by an N-terminally fused signal peptide (SP). The nascent and unfolded polypeptide is directly secreted, prior to spontaneous folding. Subsequently, the SP gets cleaved off outside of the cell, the protein is folded and released into the growth medium (Freudl 2018). For *B. subtilis* and others, it has been demonstrated that the combination of a specific SP with a certain recombinant protein determines the efficiency of the overall secretion process which cannot be predicted (Brockmeier et al. 2006; Freudl 2018; Hemmerich et al. 2016; Mathiesen et al. 2008). The secretion of proteins using the SEC-dependent pathway provides an excellent route if a recombinant protein is known to form insoluble inclusion bodies intracellularly (Freudl 2018). For *B. subtilis* 173 SEC-dependent SPs were described (Brockmeier et al. 2006). A similar number of SEC-dependent SPs was identified for *P. megaterium* DSM319 (www.megabac.tu-bs.de; Hiller et al. 2004). The efficiency of seven *P. megaterium* SPs on secretion was evaluated using the heterologous *Thermobifida fusca* hydrolase, resulting in highly variable levels of secreted protein (Stammen et al. 2010a) (Fig. 3). A later study found similar effects when looking at the secretion of the endoglucanase EGI1 and the multimodular cellulase Cel9AT with five (for EGI1) and four (for Cel9AT) different SPs, respectively, tested (Kalbarczyk et al. 2018). Furthermore, some studies show secretion of recombinant proteins using their original SP including the dextransucrase DsrS from *Leuconostoc mesenteroides* (Malten et al. 2005a), penicillin G acylases from different *Bacillus* species (Mayer et al. 2019; Yang et al. 2006), and a keratinase from *B. licheniformis* (Radha and Gunasekaran 2007; Radha and Gunasekaran 2008) (Tab. 1). For most secretion experiments, the *P. megaterium* strain MS941, a DSM319 variant lacking the gene coding for the extracellular neutral metalloprotease NprM, was used as this strain reveals a reduction of 98.5% of extracellular protease activity (Wittchen and Meinhardt 1995).

### Further adaptations of the recombinant plasmid system in *P. megaterium*

Subsequently, to enable one-step protein purification, various tag-based affinity chromatography methods can be used (Terpe 2003). Thus, the *P. megaterium* plasmid systems were designed to create a plethora of N- and/or C-terminal fusion with His_6_- or StrepII-affinity tags in combination with protease cleavage sites for tag removal (Biedendieck et al. 2007c). When combined with extracellular protein production, affinity tags provide rapid tools for protein purification, especially along with continuous cultivation processes (Gädke et al. 2017a; Gädke et al. 2017b). Moreover, a codon plus system employing the co-expression of genes for tRNAs with rare codons often found in heterologous target genes showed a general positive effect on recombinant protein production by *P. megaterium* (Finger et al. 2015) (Fig. 3). For genomic modifications, a plasmid system based on a temperature sensitive promoter was established allowing gene integration and deletion (Biedendieck et al. 2011). Just recently, the group of Hannemann developed a CRISPR-Cas9 system for the genome editing of *P. megaterium* with an efficiency up to 100% (Hartz et al. 2021).

## Production of biotechnological important proteins: multiple cytochrome P450 suitable for whole-cell transformations

P450 enzymes are frequently employed in metabolic reactions to catalyze challenging chemical reactions (Pochapsky 2020). They use a broad range of structurally diverse substrates and form their products with high stereo- and regio-selectivity. Humans carry 57 different P450 (CYP) enzymes involved in the metabolism of steroid hormones, other sterols, vitamin D_3_, eicosanoids, fatty acids, and retinoic acid (Luo and Liu 2020; Rendic and Guengerich 2021; Sarparast et al. 2020). They catalyze almost exclusively monooxygenase reactions through the activation of molecular oxygen using a single electron. The major type of reaction is the hydroxylation of difficult to activate C-H bonds. For this purpose, they contain a heme group as single-electron transfer agent. Consequently, catalyzed reactions require electron donors like NADH or NADPH. Flavin or iron-sulfur proteins transfer electrons from NADH or NADPH to the P450-bound heme (Chiliza et al. 2020; Li et al. 2020).

Since most bacterial and fungal P450s are cytosolic and soluble, these variants are better suited for biotechnology applications compared to their membrane-bound plant and mammalian homologs (Distefano et al. 2021; Finnigan et al. 2020; Iizaka et al. 2021; Toplak et al. 2021; Zhang et al. 2021b). In *P. megaterium* a number of different P450 enzymes were found. Seven cytosolic P450s (CYP) from different *P. megaterium* strains have been described. CYP106A2 from strain ATCC13368 (Berg et al. 1976; Schmitz et al. 2018) and the CYP109E1 from strain DSM319 (Jóźwik et al. 2016) rely on a FAD-dependent ferredoxin reductase and a corresponding ferredoxin as electron donor and transfer proteins. The best studied CYP102A1 from strain DSM32, also known as cytochrome P450-BM3, consists of an N-terminal P450 domain followed by a flavodoxin and a reductase domain. Therefore, this in vitro system is self-sufficient and channels electrons directly from NADPH, which accelerates monooxygenase rate in comparison to other P450s (Cook et al. 2016; Miura and Fulco 1974; Whitehouse et al. 2012). The remaining four *P. megaterium* P450s all require an external redox partner. These include CYP106A1 from strain DSM32 (He et al. 1989; Lee et al. 2015) and from strain DSM319 (Brill et al. 2014), CYP109A2 from strain DSM319 (Abdulmughni et al. 2017a), and CYP107DY1 from strain QM B1551. Interestingly, CYP107DY1 is the first plasmid encoded P450 found in *Bacillus* species. Since no CYP107 homologs can be found in the genomes of *P. megaterium* strains, CYP107DY1 may have been obtained by horizontal gene transfer. This observation suggests a possible role of P450s in the adaptation and even evolution of bacteria (Milhim et al. 2016).

### Whole-cell systems for recombinant production of P450s in *P. megaterium*

To study P450s, this is typically performed using *E. coli* recombinant expression and studied in vitro with corresponding electron transfer proteins and cofactors. However, in vitro substrate conversion rates are often limited, which may constitute a problem for large-scale industrial applications. The use of whole-cell systems can overcome these in vitro limitations, although the import of the substrates and the export of the product might also be limited (Bernhardt and Urlacher 2014). Whole-cell systems have been biotechnologically employed for CYP106A1 from *P. megaterium* strain DSM319, CYP106A2 from *P. megaterium* strain ATCC13368 (Bleif et al. 2010; Bleif et al. 2012), and CYP109A2 (Abdulmughni et al. 2017a) and CYP109E1 (Abdulmughni et al. 2017b) from strain DSM319, all using *P. megaterium* as a production host (Tab. 1). In addition, *E. coli*-based whole-cell systems using CYP107DY1 from *P. megaterium* strain QM B 1551 (Milhim et al. 2016) and CYP102A1 from strain DSM32 (Chu et al. 2016) have been reported. Finally, membrane-bound mammalian P450 CYP11A1 was recombinantly produced in *P. megaterium* (Stenger et al. 2018).

## Production of recombinant proteins: the *P. megaterium* cell-free transcription-translation system

For future progress with *P. megaterium*, a novel cell-free transcription-transcription tool was recently developed to study fundamental molecular biology and accelerate the testing of gene expression systems (Moore et al. 2018). Within synthetic biology, there has been a renewed interest in cell-free transcription-translation systems (Tinafar et al. 2019; Cole et al. 2020). Cell-free systems require a cell extract, energy solution, and plasmid DNA to synthesize recombinant proteins. These reactions can be performed in the microscale range (nL to mL range) either in test tube reactions, microtiter (96, 384, 1536 well format) plates, or microfluidics (Laohakunakorn et al. 2020). While *E. coli* remains the dominant cell-free system, a range of new cell-free systems has recently been developed from other major prokaryotic expression systems, including *B. subtilis*, *Streptomyces* spp., *Clostridium autoethanogenum*, *Pseudomonas putida*, and *Vibrio natriegens* (Gregorio et al. 2019; Cole et al. 2020). In terms of protein yield, although many systems are still in development, cell-free protein production is often monitored with GFP as a standard. For this, *P. megaterium* (134 ng μL^−1^ GFP) compares favorably to other popular Gram-positive hosts such as *B. subtilis* (21.6 ng μL^−1^) and *Streptomyces lividans* 66 (~100–400 ng μL^−1^) (Cole et al. 2020).

For *P. megaterium* DSM319, an optimized cell-free protocol (also active in DSM509) was recently developed (Moore et al. 2018). One key advantage of cell-free systems is the study of the biological numbers that underpin protein synthesis in combination with computational prediction models (Moore et al. 2018). A key finding of the *P. megaterium* cell-free system was potential rate-limiting steps in protein synthesis. For example, under the conditions studied, maximal translation rates were an order of magnitude slower than that of *E. coli* cell-free (Garamella et al. 2016).

In addition, the kinetics of the xylose-inducible promoter system (see above) were characterized in detail. For example, the dissociation constant for XylR and operator binding was determined at 12.9–14.2 nM with a Hill coefficient of 1.74–1.8. Finally, as an example of the power and speed of cell-free systems, using a liquid handling robot, up to 500 *P. megaterium* plasmids with varying promoter and RBS regions were rapidly screened for activity within 24 h. This permits the rapid characterization of DNA plasmid designs for forward engineering in *P. megaterium* cells (Moore et al. 2018). In summary, this recent development provides a high-yield and rapid cell-free tool for the study and engineering of *P. megaterium* for future metabolic engineering and synthetic biology applications.

## Whole-cell applications: *P. megaterium* as a plant growth-promoting bacterium

Recently, within the last 20 years, scientists have discovered that the plant microbiome is essential for the survival of plants in changing environmental conditions including invasion by pathogenic microorganisms and insects (Berger and Gutjahr 2021; Genre et al. 2020; Haskett et al. 2020; Ortiz and Sansinenea 2021; Prsic and Ongena 2020; Vishwakarma et al. 2020; Zhang et al. 2021a). In conclusion, the health and growth of cultural plants can be influenced by the composition of its root and leave microbiome (Ray et al. 2020). In recent years, the beneficial effect of *P. megaterium* on plant growth has become a growing matter of interest. It has been described for a number of different plants including the model organism *Arabidopsis thaliana* (López-Bucio et al. 2007; Ortíz-Castro et al. 2008), the commercially important plants tomato (*Solanum lycopersicum*) (Ibort et al. 2017; Porcel et al. 2014), tea (*Camellia sinensis*) (Chakraborty et al. 2006), maize (*Zea mays*) (Al-Enazy et al. 2017; Marulanda et al. 2010), mustard (*Brassica juncea* L.) (Kang et al. 2014; Rajkumar and Freitas 2008), rice (*Oryza sativa* L.) (Feng et al. 2017), bean (*Phaseolus vulgaris*) (Korir et al. 2017; Ortíz-Castro et al. 2008), soybean (*Glycine max*) (Zhou et al. 2017), and oilseed rape (*Brassica napus*) (Hu et al. 2013). Three different mechanisms of plant growth promotion by *P. megaterium* have been described.

The first role can be summarized as biofertilizer. Phosphorus is essential for plant growth; however, its bioavailable form is often present in very low amounts (Liu 2021). The transformation of phosphorus in minerals and organic sources to their bioavailable forms occurs through secretion of organic acid in combination with acid phosphatases and phytases (Kang et al. 2014; Martínez-Viveros et al. 2010). *P. megaterium* secrets organic acids providing the main basis of phosphate biofertilization using secreted acid phosphatase and phytases (Hu et al. 2013). In addition, *P. megaterium* can provide reduced nitrogen to plants (Ding et al. 2005; Liu et al. 2006; Singh et al. 2020). Currently, a number of different *P. megaterium* fertilizer preparations often in combination with other bacteria are commercially available by different manufacturers for large-scale agricultural applications. These combinations including *P. megaterium* are also subjects of different patents. Wang et al. claim the release of potassium and phosphorus, the fixation of nitrogen, the inhibition of harmful bacteria in the soil, and the prevention of different diseases using a mixture of *P. megaterium*, further *Bacillus* strains and Gram-negative bacteria (Wang et al. 2009). Recently a fertilizer and its preparation was patented for the hydrolysis of phosphorus containing only *P. megaterium* but combined with organic matter and ammonium sulfate (Jianzhong 2019). 

Secondly, plant growth-promoting bacterium can also be responsible for changes in environmental concentrations of phytohormones and other regulators of plant growth. Interestingly, the production of the auxin indole acetic acid (IAA) by *P. megaterium* resulted in a plant growth-promoting effect, as reported repeatedly for different plants (Chakraborty et al. 2006; Feng et al. 2017). Furthermore, the cultivation of *A. thaliana* with *P. megaterium* lead to higher concentrations of the isoprenoid plant hormone abscisic acids (ABA) in plant leaves, thus improving drought stress tolerance (Zhou et al. 2016). It was postulated that secretion of the polyamine spermidine by *P. megaterium* was responsible for the upregulation of the expression of ABA-associated genes and subsequent production of ABA in the plant. Ortíz-Castro et al. reported that cytokinin signaling plays a central role in the plant growth-promoting effect by *P. megaterium* on *A. thaliana* under defined lab conditions (Ortíz-Castro et al. 2008). In addition to the classical phytohormones, the role of so-called volatile compounds as plant growth-promoting substances is recently gaining more interest (Ryu et al. 2003; Sharifi and Ryu 2018). For *P. megaterium* the positive effect of the volatile compound 2-pentylfuran on the growth of *A. thaliana* has been observed (Zou et al. 2010). The mechanism by which this compound promotes plant growth is still unknown. Finally, acetoin produced by a *P. megaterium* strain promoted the growth of *A. thaliana* (Ryu et al. 2003).

Thirdly, *P. megaterium* can act as a biopesticide or biocontrol agent. The multiple anti-pathogenic mechanisms of *P. megaterium* are divers. An antifungal activity against the tea pathogen *Fomes lamaoensis*, the cause of brown root rot, is possibly related to the production of iron-chelating siderophores. Additionally, the enhanced secretion of the plant defense-related peroxidase, phenylalanine ammonia lyase, chitinase, and β-1,3-glucanase by *P. megaterium* was observed (Chakraborty et al. 2006). These enzymes are postulated to act directly against the fungal cell wall, thereby protecting the plant. The secretion of chitinase, β-1,3-glucanase, and protease by *P. megaterium* also mediated a protective effect against the fungus *Rhizoctonia solani*, the causative agent of “damping-off”, a destructive disease of plant seedlings, and against root rot in tomato (*Lycopersicon esculentum* Mill) (Solanki et al. 2012). The treatment of *R. solani-*caused diseases with *P. megaterium* strain ATCC55000 was patented in 1995 where its function as a biological control agent was described but lacks explanation. Moreover, the additional role of strain ATCC55000 in stimulating growth and yield in soybeans was part of the invention (Liu and Sinclair 1995). Another antifungal property of *P. megaterium* mediated by an unidentified volatile compound was found against the aflatoxin-producing *Aspergillus flavus* found on rice grains (Mannaa et al. 2017). A mixture of the three rhizobacterial bacteria *P. megaterium*, (*Peri*)*Bacillus simplex*, and *Sinorhizobium fredii* coated on soybean seeds revealed a clear protective effect against the “soybean cyst nematode” (*Heterodera glycines*) (Zhou et al. 2017).

Finally, coming back to the *P. megaterium* P450s, CYP102A1 (P450-BM3) most likely plays an important role in the regulation quorum sensing by soil bacteria through the inactivation of acyl homoserine lactones (AHLs). These molecules are known signaling molecules in the communication of Gram-negative bacteria (Chowdhary et al. 2007). Interestingly, the ability of *P. megaterium* to degrade AHLs suggests a link of their plant-protective, quorum-quenching activity to the quorum sensing of plant pathogenic bacteria (Dong et al. 2001). In summary, *P. megaterium* revealed a whole variety of molecular strategies of plant growth-promoting effects.

## Conclusion and perspectives

*P. megaterium* is a fast-growing giant cell factory, with past and current industrial applications, and a promising alternative to standard model organisms (Eisenstein 2016). In 1994, Patricia S. Vary wrote “Prime time for *Bacillus megaterium*” (Vary 1994) which was followed 13 years later by the update “*Bacillus megaterium* - from simple soil bacterium to industrial protein production host” (Vary et al. 2007). Now, 14 years later, we have a new name for our well-known bacterium, while there are more than 20 fully sequenced genomes, a commercialized recombinant plasmid toolkit for high-yield recombinant protein production to the g per L scale, and a first cell-free transcription-translation in vitro system. In addition, our knowledge of whole-cell systems for the production of important P450 enzymes and also as a plant growth-promoting bacterium, the use in the production of bioplastics, and a prominent role in the understanding of B_12_ biosynthesis increased significantly.

So, what is needed next to develop *P. megaterium* into a competitive biotechnological production host? To support rapid and more straightforward biotechnological research, a complete genome-level single-gene knock-out library will provide an accurate picture of all non-essential genes, as is available for many model microbes. This is desirable to identify genes that may limit/benefit questions relating to recombinant protein production, metabolic capacity, or self-regulatory processes. Although the transformation of *P. megaterium* protoplasts is completely sufficient for the introduction of single plasmids, to test entire gene banks in this organism, a better transformation system is also needed. Since almost all genes necessary for the formation of natural competence are present in *P. megaterium*, an easier integration of DNA should be possible, which will serve as another important developmental feature. In addition, due to its natural size, *P. megaterium* represents a perfect tool for cell biological studies. Combining time-lapse microscopic studies with corresponding bioinformatic tools and modeling approaches is of interest (Münch et al. 2015).
